# Effect of the Crosslinker Introduction Stage on the Structure and Properties of Xanthan Gum–Acrylamide Graft Copolymer

**DOI:** 10.3390/polym17212841

**Published:** 2025-10-24

**Authors:** Anton K. Smirnov, Diana F. Pelipenko, Sergei L. Shmakov, Andrey M. Zakharevich, Anna B. Shipovskaya

**Affiliations:** 1 Institute of Chemistry, Chernyshevsky Saratov State University, 83 Astrakhanskaya St., 410012 Saratov, Russia; diana21062010@mail.ru (D.F.P.); shmakovsl@info.sgu.ru (S.L.S.); shipovskayaab@yandex.ru (A.B.S.); 2 Education and Research Institute of Nanostructures and Biosystems, State University, 83 Astrakhanskaya St., 410012 Saratov, Russia; lab-15@mail.ru

**Keywords:** xanthan gum, acrylamide, microwave-assisted synthesis, graft copolymer, structure, sorption

## Abstract

Graft copolymers of polysaccharides with side chains of carbon-chain monomers have significant potential for a variety of practical applications. In this work, the effect of the *N*,*N*-methylene*bis*acrylamide (MBA) introduction stage and acrylamide concentration in microwave-assisted radical copolymerization with xanthan gum on the structure and sorption properties of the cross-linked graft copolymer was studied. It has been found that the spatial network density and average molecular weight of interstitial fragments can be controlled by varying these factors. Moderate crystallinity (<50%) and a highly developed surface of our synthesized samples were revealed using XRD and SEM. The graft copolymer exhibits the Schroeder effect; its liquid water sorption obeys Fick’s law and increases with MBA introduction at later stages and with increasing grafting degree, reaching 17.2 g/g. Studying the methylene blue sorption kinetics using pseudo-first/pseudo-second order models, a combined model and an average pseudo-order model have shown that the lower the monomer concentration in the reaction mixture and the earlier (from the onset of the reaction) the cross-linking agent is introduced, the higher the equilibrium sorption. The observed “equilibrium degree of sorption on xanthan gum vs. pseudo-order” relationship, which passes through a minimum, is explained by chemisorption and the sorbate consumption effect. An assumption is made about the prospects of using our synthesized copolymers for designing selective sorbents and ion-exchange membranes.

## 1. Introduction

A promising direction for the functionalization of natural polysaccharides is the synthesis of graft copolymers by graft polymerization of monomer units of various chemical natures onto a polysaccharide matrix. For example, the combination of a polysaccharide backbone with carbon-chain side chains allows the design of materials for a wide range of practical applications: from medical and biological sciences to the oil industry [1,2,3]. The multifunctionality of such graft copolymers is due to the synergistic combination of biodegradability and biocompatibility of the natural matrix with high mechanical strength and chemical resistance of the synthetic component [4,5,6]. Of particular interest are graft copolymers based on xanthan gum (XG), a bacterial polysaccharide with macromolecules containing two types of reactive groups (hydroxyl and carboxyl) [7,8]. Their key advantages also include the formation of highly viscous solutions at low xanthan gum concentrations and pH-dependent changes in the hydrodynamic volume of macrochains without their chemical degradation [9].

A current trend in the functional modification of xanthan gum is the creation of spatially cross-linked macromolecular structures with side branches of polyacrylamide and its derivatives [10,11,12]. Such graft copolymers are synthesized via the mechanism of radical polymerization of acrylic monomers to a growing active center [13]. Spectroscopic studies have shown that the primary active centers are initiated predominantly on the polysaccharide chain as a result of proton abstraction from the primary hydroxyl group at C6 of the substituted glucopyranose unit [14]. The reaction is usually carried out in an inert atmosphere to minimize oxidative–destructive side processes. A competing homopolymerization reaction proceeds in parallel with the graft copolymerization, whose product is removed during the purification stage. Kinetic chain growth completion occurs due to monomer exhaustion or recombination of macroradicals in growing side chains. Quantitative evaluation of the process efficiency traditionally involves calculating the degree and efficiency of grafting [15].

To date, graft copolymers of xanthan gum with acrylamide [16,17] and acrylic acid [18,19], as well as block polymer acrylic side chains [20,21,22] have been synthesized and studied. The classical approach to their synthesis involves the use of potassium or ammonium persulfates as initiators and N,N-methylene*bis*acrylamide (MBA) as the primary crosslinking agent [7,18,22]. In some studies, trimethylolpropane triglycidyl ether [17,23] and *N*-isopropyl acrylamide [24] were used as functional alternatives to MBA.

The mass ratio of the polymerization reagents is a key parameter influencing both the synthesis efficiency and the graft copolymer properties. The highest grafting parameters have been achieved for xanthan gum/monomer compositions in the mass range of 1:5–1:45 [25,26]. Such graft copolymer samples are also characterized by the best water sorption indices, reaching ~12 g/g [16,27]. This opens up the prospect of producing water-retaining agents, sorbents of cationic pollutants, and carrier matrices for targeted drug delivery on their basis [18,28]. However, deviations from the optimal range have been experimentally proven to lead to a decrease in yield and deterioration in the quality of the target product [22,23].

Graft copolymerization of xanthan gum with acrylic monomers is traditionally carried out at 70–80 °C for 3–4 h under nitrogen flow. This process typically results in partial degradation of the polysaccharide matrix. The use of microwave (MW) irradiation eliminates this drawback and reduces the time to obtain the grafted product down to 3–4 min [16,29]. This approach creates the required temperature conditions in the reaction mixture almost instantly, eliminates the need for an inert medium, and significantly increases the rate and yield of the copolymer [16,19,25]. In addition, the use of MW exposure improves the structural and compositional homogeneity of the resulting graft copolymers [30].

**Scheme 1:**
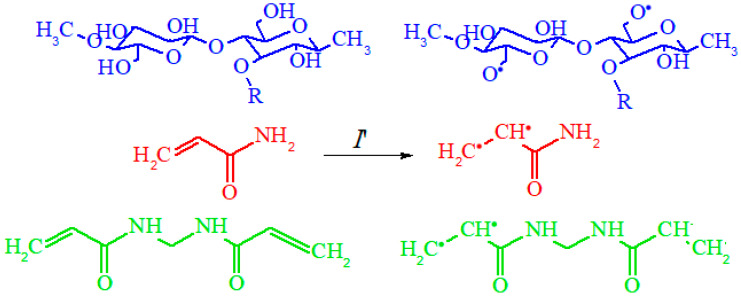
Possible localizations of unpaired electrons on the radical atoms of XG, AAm and MBA molecules at the initiation stage of radical graft polymerization, *I*′ is an initiator radical.

The need to add all reagents to the reaction mixture before the process begins is a significant limitation of known methods for graft polymerization of acrylic monomers onto xanthan gum, which leads to three types of radicals being formed during the initiation stage [17,22], e.g., in the frequently studied polymerization of acrylamide (AAm) onto xanthan gum using MBA, in addition to the abovementioned macroradical at C6 of the structural units of the macromolecular component, radicals of the monomer and cross-linking agent are generated (Scheme 1). This leads to the chaotic progression of multiple competing reactions. In particular, MBA biradicals may crosslink both growing polyacrylamide chains and fragments of individual or adjacent xanthan gum macromolecules. Growing side chains could also be attached to the polysaccharide chain not only by grafting the AAm radical onto C6–O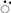 of the main chain, but also by means of a crosslinking bridge from MBA [10,20,31]. Thus, the activation of additional reaction centers leads to a statistical nature of crosslinking and, consequently, to the formation of a spatial network with an uncontrolled architecture.

We here propose introducing the crosslinking agent at different stages of radical copolymerization as an approach to controlling the dislocation of crosslinking sites and, consequently, obtaining a graft copolymer with a desired macromolecular architecture, e.g., if MBA is introduced into the reaction mixture at the chain growth stage, crosslinking of the growing side branches will be most likely. Introducing MBA into the system after the side chain growth completion, i.e., at the initial stage of termination, should largely promote the formation of crosslinks involving macromolecular xanthan gum fragments. In this case, crosslinking of the kinetic chains of polyacrylamide would be insignificant. If our hypothesis is correct, then it will be possible to specifically synthesize graft copolymers with certain properties required for a specific application by varying the crosslinker introduction stage.

The purpose of this study was to evaluate the effect of introducing the cross-linking agent *N*,*N*-methylene*bis*acrylamide during the radical copolymerization of acrylamide on xanthan gum on the localization of cross-linking sites, the spatial network architecture, and the structure and sorption properties of the cross-linked graft copolymer.

## 2. Materials and Methods

### 2.1. Materials

The study used an XG sample with an average viscosity molecular weight 1700 kDa (TNN group DL, Dalian, China), 40% aqueous solution of AAm and MBA powder (ACRYPOL Ltd., Saratov, Russia), ammonium persulfate (APS) (Hebei Fiza Technology Co., Ltd., Shijiazhuang, China), 95% ethyl alcohol and isopropyl alcohol (VEKTON Corp., St. Petersburg, Russia), methylene blue (Moskhimfarmpreparaty named after N.A. Semashko, Moscow, Russia). All chemical reagents were of chemically pure grade and were used without additional purification.

### 2.2. Preparation of Solutions

A 1% solution of the polysaccharide (pH ~ 7) was prepared in a 150 mL flask. A 1.2 g sample of xanthan gum was dispersed in 2 mL of ethanol for 1 min by gently shaking the vessel, the calculated volume of distilled water was added and heated 10 times in a Monowave 200 microwave reactor at 700 W (Anton Paar, Graz, Austria) for 10 s (without bringing to boiling) until the ethanol evaporated and the polymer was visually dissolved.

A 1% solution of the initiator and a 3% solution of the cross-linking agent were prepared in 50 mL flasks by dissolving APS and MBA, respectively, in distilled water using a standard method.

### 2.3. Microwave-Assisted Synthesis of Graft Copolymer

A 50 mL reaction flask was charged with 12 mL of the 1% XG solution, 3 mL of the 1% APS solution and a given volume (2–8 mL) of the 40% AAm solution. The composition of the polymerization mixture (XG and AAm) was expressed by the mass ratio *m*_XG_:*m*_AAm_ (Table 1).

The graft copolymer was synthesized in three ways. In the first one, MBA was introduced into the reaction system simultaneously with XG, AAm and APS, i.e., at the initiation stage (stage *t_i_*). The reaction mixture was microwaved in a Monowave 200 reactor (Anton Paar, Graz, Austria) at 700 W for 3 min. In the second option, the cross-linking agent was introduced at the chain growth stage (stage *t_p_*). First, the reaction mixture of XG, AAm, and APS was microwaved for 1 min, then MBA was added, and the MW reaction was continued for another 2 min. In the third option, the cross-linking agent was introduced at the end of the chain growth stage (stage *t_f_*), considered as the initial stage of macroradical termination. The polymerization mixture of XG, AAm, and APS was microwaved for 3 min, MBA was added, and the polymerization mixture was stirred. The time range of our synthesis was selected based on literature data [26].

In each option, after the MW-assisted synthesis was completed, the reaction product was cooled down to room temperature and the homopolymer and monomer residues were removed by washing with a water–ethanol 2:8 mixture until the washing waters became visually transparent. The graft copolymer was precipitated by keeping it in the above water–alcohol mixture at a temperature of 4 °C for 48 h. The product was dried in a heating cabinet at 70 °C until an air-dried monolith sample of constant weight was formed. The monoliths were ground in a porcelain mortar to a powder state. Graft copolymerization samples were designated as XG-*g*-PAAm-No, where the number corresponds to “sample No.” in Table 1.

### 2.4. Research Methods

Gravimetric measurements were performed on an Ohaus Adventurer AR1530 scale (Shanghai, China), weighing accuracy ± 0.002 g.

FTIR spectra were recorded on an FSM-1201 IR Fourier spectrometer (Infraspek LLC, St. Petersburg, Russia) in a wavenumber range of 500–4000 cm^−1^ in KBr tablets. The spectra were decoded using spectral tables [32].

X-ray diffractometry (XRD) was performed on a DRON-8T multifunctional X-ray diffractometer (JSC “IC Burevestnik”, St. Petersburg, Russia) with CuK_α_ radiation using a Goebel parabolic mirror (AXO Dresden GmbH, Dresden, Germany) and a Mythen 2R1D position-sensitive detector with 640 channels (Dectris, Baden, Switzerland) and a resolution of 2θ = 0.0144 degs. The degree of crystallinity was estimated using the method described elsewhere [33].

SEM images were obtained on a MIRA II LMU microscope (TESCAN, Brno, Czech Republic) at a voltage of 30 kV and a conductive current of 400 pA. A 5 nm thick gold layer was applied to each sample using a K450X CarbonCoater (Emitech Ltd., Chelmsford, England).

### 2.5. Physicochemical Characterization of the Graft Copolymer

The degree of grafting was calculated using Equation (1):

(1) $$G=\frac{m_{1}-m_{0}}{m_{0}}\cdot 100\%,$$

where *m*_0_ and *m*_1_ are the masses of the polysaccharide taken into the reaction and the synthesized graft copolymer, respectively.

The swelling kinetics and the sorption degree of liquid water (water absorption capacity) were estimated by the immersion method, keeping the samples in H_2_O (sorbent/sorbate weight ratio 1:100) at 22 ± 2 °C for 44 h. The sorption degree of water vapor was estimated by the desiccator method at the same temperature for 54 h [34]. The equilibrium water absorption (*W*_im_, g/g) and the sorption degree of H_2_O vapor (*W*_vap_, g/g) were calculated based on the ratio of the masses of the swollen and initial graft copolymer samples, respectively.

The polymer density (*ρ_c_*, g/cm^3^) was estimated gravimetrically. A weighed portion of each graft copolymer sample (~0.5 g) was placed into a 10 mL pycnometer filled to the mark with isopropyl alcohol and brought to constant weight; the excess liquid was removed, and the sample was weighed. The density, the polymer fraction in the swollen graft copolymer network (*f*), and the molecular mass of the internodal regions of the graft copolymer samples (*M_c_*, g/mol) were calculated using Equations (2)–(4), respectively:

(2) $$\rho_{c}=\frac{m_{c}\cdot \rho_{i}}{m_{p}\;+\;m_{c}\;-\;m_{p+c}}$$

(3) f =mdρcmdρc+Vr

(4) Mc=− ρcVp(f13−f2)ln(1 −f) +f +χF–Hf 2

where *m_c_* and *m_d_* are the masses of the sample portion for determining density and water absorption, respectively, g; *ρ_i_* is the density of isopropyl alcohol, 0.785 g/cm^3^; *m_p_* and *m_p_*_+*c*_ are the masses of the pycnometer with isopropyl alcohol without and with the weighed sample, respectively; *V_r_* the absorbed water volume, cm^3^; *V_p_* the partial volume of water, cm^3^/mol; *χ*_F–H_ the Flory–Huggins parameter, which, according to the additivity principle, was considered proportional to the molar fractions of the components in the copolymer. For xanthan gum *χ*_F–H_ = 0.82 [35], for polyacrylamide *χ*_F–H_ = 0.48 [36]; for samples XG-*g*-PAAm-1 (-2, -3) χ_F–H_ = 0.52, for samples XG-*g*-PAAm-4 (-5, -6) − χ_F–H_ = 0.50, for samples XG-*g*-PAAm-7 (-8, -9) − χ_F–H_ = 0.49 (see Table 1 for sample numbering).

Sorption capacity was estimated spectrophotometrically using a B-1100 VEK 2109010 spectrophotometer from Shanghai Mapada Instruments Co., Ltd. (Shanghai, China) at *λ* = 660 nm by keeping samples in a 10^−4^ mol/L methylene blue solution at 22 ± 2 °C for 32 h. The residual dye concentration in the solution was determined using a pre-built calibration line. The sorption capacity per copolymer (*Qe*, mg/g) was calculated using Equation (5):

(5) $$Qe\;=\;\frac{(C_{0}\;-\;C_{e})\cdot V\cdot M\cdot 1000}{m},$$

where *C*_0_ and *C_e_* are the dye concentrations before and after sorption, respectively, mol/L; *V* is the volume of the solution from which sorption was carried out, L; *M* is the molar mass of methylene blue, 320 g/mol; *m* is the mass of the sorbent, g; 1000 is the g→mg converting factor. The sorption capacity per xanthan gum (*Qe*_XG_, mg/g) was calculated using Equation (6):

(6) $${\mathrm{Qe}}_{\mathrm{XG}}=\;\mathrm{Qe}\left(\frac{G}{100}+1\right)$$

To describe the methylene blue sorption kinetics *Qe* = *f*(*t*), the kinetic models of pseudo-first (7) and pseudo-second order (8), as well as the intraparticle diffusion model (9) [37] were used:

(7) $$\ln\left({\mathrm{Qe}}_{\max}-{\mathrm{Qe}}_{t}\right)\;=\;\ln{\mathrm{Qe}\prime }_{\max}-K_{1}\cdot t,$$

(8) $$\frac{t}{{\mathrm{Qe}}_{t}}=\frac{1}{K_{2}\cdot {\mathrm{Qe}\prime }_{\max}^{\;2}}+\frac{t}{{\mathrm{Qe}\prime }_{\prime }},$$

(9) $${\mathrm{Qe}}_{t}=K_{i}t^{1/2}+I$$

where *Qe*_max_ and *Qe*′_max_ are the equilibrium experimental and theoretical sorption capacities of the sample, respectively, mg/g; *Qe*_t_ is the sorption capacity at time *t*, mg/g; *t* is time, min; *K*_1_ is the rate constant of pseudo-first order kinetics, min^−1^; *K*_2_ is the rate constant of pseudo-second order kinetics, (g/mg·min); *K*_i_ is the rate constant of intraparticle diffusion (mg/g·min^1/2^); and *I* is a parameter proportional to the sorption layer thickness (mg/g).

The determining contribution of physical (pseudo-first order) or chemical sorption (pseudo-second order) was judged by the most accurate match of our experimental data with the corresponding model [38,39]. The limiting contribution of boundary or intraparticle diffusion of the sorbate was estimated by the slope of the linear sections of the dependence *Qe* = *f* (*t*^1/2^).

We also performed calculations according to the combined model, Equations (10) and (11) [40], which takes into account both kinetic pseudo-orders, as well as the pseudo-*n*-order model to generalize both models, Equations (12) and (13):

(10) $$\frac{d{\mathrm{Qe}}_{t}}{dt}\;={\;K}_{1}\left({\mathrm{Qe}}_{\max}-{\mathrm{Qe}}_{t}\right)\;+\;K_{2}{\left({\mathrm{Qe}}_{\max}\;-\;{\mathrm{Qe}}_{t}\right)}^{2}$$

(11) $${\mathrm{Qe}}_{t}={\;\mathrm{Qe}}_{\max}\left(1\;-\;\frac{1}{\left(K\cdot {\mathrm{Qe}}_{\max}+1\right)\exp\left(K_{1}t\right)-K\cdot {\mathrm{Qe}}_{\max}}\right)\;={\;\mathrm{Qe}}_{\max}\left(1\;-\;\frac{1}{\left.K\cdot {\mathrm{Qe}}_{\max}\right.\left[\exp\left(K_{1}t\right)-1\right]+\exp\left(K_{1}t\right)}\right)$$

(12) $$\frac{d{\mathrm{Qe}}_{t}}{dt}={\;K}_{n}{\left({\mathrm{Qe}}_{\max}-{\mathrm{Qe}}_{t}\right)}^{n}$$

(13) $${\mathrm{Qe}}_{t}={\;\mathrm{Qe}}_{\max}\left[1\;-{\;\left(1+(n-1)\cdot K_{n}{\mathrm{Qe}}_{\max}^{n-1}t\right)}^{\frac{1}{1-n}}\right]$$

where *K* = *K*_2_/*K*_1_.

The residual sum of squares was minimized in the mathematical package MAXIMA.

The significance of the coefficients ∆*a* and ∆*b* of the linear regression *y* = *a* + *bx* for the pseudo-first/pseudo-second models was calculated by the usual formulae: ∆*a* = ±*t*_0.95,f_ · *S*_a_ and ∆*b* = ±*t*_0.95,f_ · *S*_b_. The variances of the coefficients *S*_a_^2^ and *S*_b_^2^ and the variance of the adequacy of linear regression *S*_ad_^2^ were calculated by standard formulae of mathematical statistics.

## 3. Results and Discussion

### 3.1. Parameters of the Synthesized Graft Copolymer

Nine samples of water-insoluble, cross-linked graft copolymer of xanthan gum with acrylamide were obtained by radical MW-assisted polymerization (Table 1). The samples differed in that the cross-linking agent was introduced into the polymerization reaction at the stage of initiation (*t_i_*), chain growth (*t_p_*) or chain termination (*t_f_*). The amount of the monomer introduced into the reaction was also varied to assess the effect of the xanthan gum/acrylamide mass ratio on the synthesis and properties of the target product.

Air-dried samples of the graft copolymer of xanthan gum with acrylamide were finely dispersed white powders. During sample preparation (grinding of the synthesized monoliths), an increased strength of the samples was visually noted with an increase in the proportion of polyacrylamide in the graft copolymer.

As expected, the degree of grafting (at equal polymerization times) increased with an increase in the mass fraction of acrylamide in the reaction system, reaching a maximum value for samples XG-*g*-PAAm-7 (-8, -9) (Table 1). The samples with higher *G* values can be assumed to be characterized by a higher number of grafted chains and/or longer polyacrylamide branches. It should also be noted that under identical polymerization conditions (i.e., at an equal *m*_XG_:*m*_AAm_ ratio), the highest degree of grafting was observed for the samples synthesized with the cross-linking agent introduction at the initiation stage (*t_i_*), apparently due to the consumption of reaction centers at later stages of the cross-linking agent introduction. MBA introduction into the reaction mixture at the chain growth (*t_p_*) or chain termination (*t_f_*) stage led to a decreased degree of grafting, but not very significantly.

### 3.2. Characterization of Samples

#### 3.2.1. FTIR Spectroscopy

**Figure 1:**
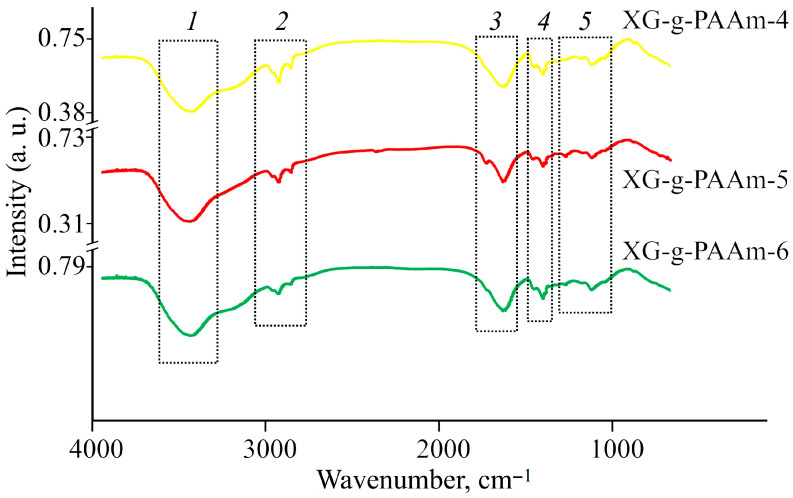
FTIR spectra of XG-*g*-PAAm-4 (-5, -6) copolymer samples obtained at a mass ratio *m*_XG_:*m*_AAm_ of 0.12:1.6. The dotted lines highlight the vibration ranges of the main characteristic signals: *1*—υ_O–H_, υ_N–H_; *2*—υ_C–H_, δ_C–H_; *3*—υ_–COO–_, _C=O_; *4*—υ_C–N_, δ_C–H_; *5*—υ_C–O, C–C, C–H_.

The FTIR spectra of XG-*g*-PAAm-4 (-5, -6) are characterized by bands in the region of 3500–3400 cm^−1^, corresponding to stretching vibrations of the N–H and O–H bonds (Figure 1, frequency range *1*). The depth and width of the signals indicate the supramolecular structure of the copolymer, characterized by a developed system of inter- and intramolecular hydrogen bonds. The signals in the range of 3000–2800 cm^−1^ are related to stretching vibrations of the bonds in the CH, CH_2_ and CH_3_ groups of xanthan gum and polyacrylamide (frequency range *2*). There are characteristic signals in the range of 1655–1630 cm^−1^, related to stretching vibrations of the C=O bonds in the ester side fragment of xanthan gum macromolecules, as well as to the amide-I of polyacrylamide (range *3*).

The bands within 1610–1600 cm^−1^ are the signals of stretching vibrations of the –COO¯ atoms of xanthan groups (Figure 1, range *3*). Superposition of the signals of deformation vibrations of the C–H bond atoms and stretching vibrations of the C–N bond gives an intense signal within 1460–1390 cm^−1^ (*4*). The bands in the frequency range of 1120–1000 cm^−1^ are related to vibrations of the C–O, C–C, and C–H bonds of the glucopyranose ring (*5*). The FTIR spectra of other samples were similar to those for XG-*g*-PAAm-4 (-5, -6) samples and are presented in the Supplementary Materials (Figure S1).

Thus, the presence of signals belonging to xanthan gum, polyacrylamide, and *N*,*N*-methylene*bis*acrylamide in the FTIR spectra confirms successful copolymerization and the formation of a graft copolymer in each obtained sample.

#### 3.2.2. X-Ray Diffractometry (XRD)

**Figure 2:**
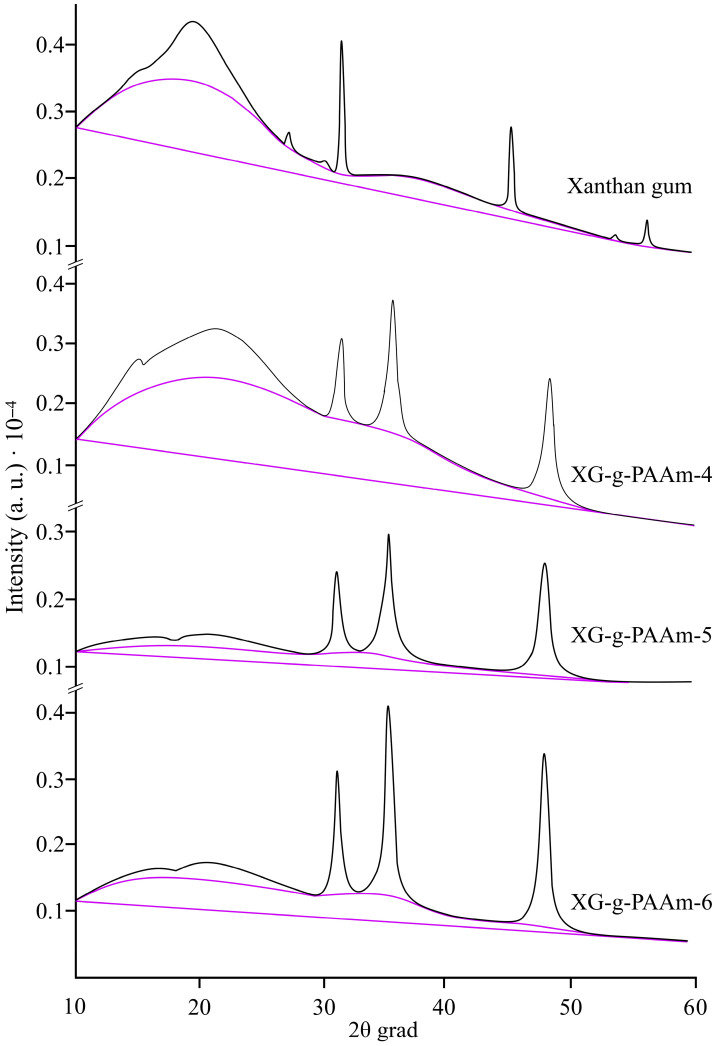
X-ray diffraction patterns of xanthan gum and XG-g-PAAm-4 (-5, -6) samples obtained at a mass ratio of *m*XG:*m*AAm 0.12:1.6. The purple lines indicate the amorphous halo.

Typical X-ray diffraction patterns of our graft copolymer, as well as the FTIR spectra, are presented using the example of XG-*g*-PAAm-4 (-5, -6) samples (Figure 2). The X-ray diffraction patterns of the other samples are presented in the Supplementary Materials (Figure S2a,b). The X-ray profiles show three diffraction peaks at 2θ~20, 32 and 45 degs, traditionally observed in the diffraction pattern of pure xanthan gum. However, there was a broadening and decrease in the intensity of the first two reflections and a shift of the third reflection towards the region of 2θ~48 degs. A new peak at 2θ~35 degs, characteristic of the X-ray diffraction patterns of polyacrylamide, was also detected for the graft copolymer. The obtained diffraction profiles of XG-*g*-PAAm-4 (-5, -6) samples indicate an amorphous–crystalline structure of the graft copolymer with a low degree of crystallinity.

The calculated crystallinity degree of the graft copolymer samples turned out to be higher than that of the initial xanthan, for which *χ* = 24.1% (Table 2). The highest and lowest values of *χ* (~30–48%) are characteristic of the samples synthesized with the cross-linking agent introduction at the *t_p_* and *t_i_* stage, respectively. The graft copolymers obtained with MBA introduction at the *t_f_* stage also show insignificant crystallinity, but the *χ* values are slightly higher compared to the samples synthesized according to the classical scheme (XG-*g*-PAAm-1 (-4, -7) − *t_i_*). Overall, the amorphous–crystalline supramolecular ordering, which exceeds that of pure xanthan gum, indicates the stability and strength of our synthesized graft copolymer samples. This should also ensure good sorption properties of the graft polymer, since the amorphous portions of the polymer participate in sorption.

#### 3.2.3. Scanning Electron Microscopy (SEM)

**Figure 3:**
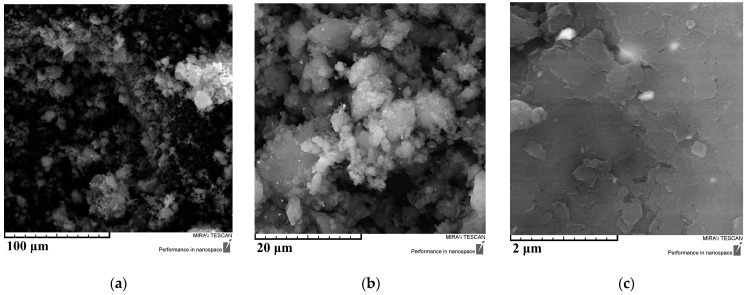

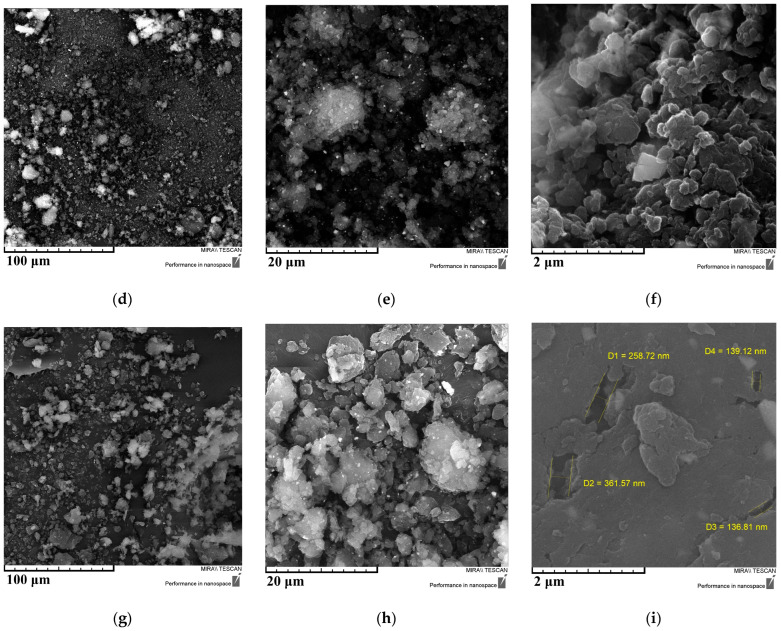
SEM images of the graft copolymer samples XG-g-PAAm-4 (**a**–**c**), XG-g-PAAm-5 (**d**–**f**), and XG-g-PAAm-6 (**g**–**i**) obtained by introducing the cross-linking agent at the chain growth stage (t_p_).

Analysis of the SEM images shows that the particles of the XG-*g*-PAAm graft copolymer have a close to spherical or ellipsoidal shape (Figure 3). The particle size varied in the range of 1–25 μm. The predominant fraction contains particles of 1–5 μm in size, making up 60–62% of the total amount of the synthesized product. Larger particles of 5–10 and 10–25 μm in size make up 20–22 and 16–20%, respectively. Roughness of the particle surface (Figure 3b,f,h,i) and the presence of microvoids of 0.1–0.8 μm in size (Figure 3c,i) are also noted. Such surface morphology of the polymer substance could have a positive effect on its sorption properties.

### 3.3. Sorption Properties

#### 3.3.1. Water Sorption

Water sorption evaluation has shown that the synthesized XG-*g*-PAAm samples exhibit high sorption capacity. In general, the process of water absorption by the graft copolymer is represented by a classical sorption curve, which includes an intense increase in the sorption degree at the initial stage (6–8 h), and a plateau at the final stage (after 35–40 h). Throughout the sorption experiment, the graft copolymer stability was visually noted, without any degradation processes.

**Figure 4:**
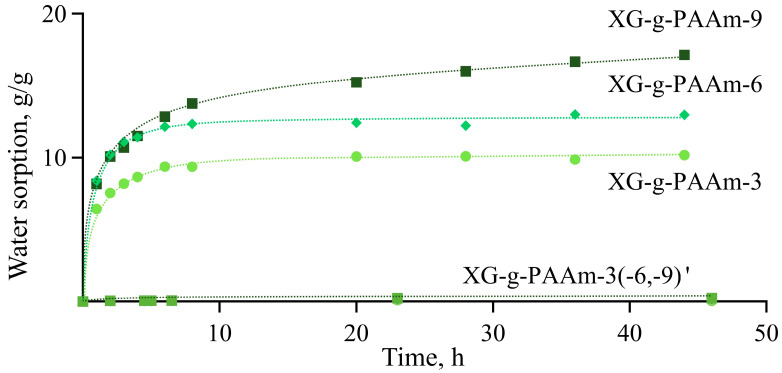
Sorption kinetics of liquid water and H_2_O vapor (marked with a prime) by XG-*g*-PAAm-3 (-6, -9) graft copolymer samples obtained by introducing the cross-linking agent at the chain termination stage (*t*_f_).

Let us consider in more detail the water absorption kinetics using the example of XG-*g*-PAAm-3 (-6, -9) samples obtained by introducing the cross-linking agent at the chain termination stage (*t_f_*), but differing in the degree of grafting and the side branch length (Figure 4). With an increase in the mass fraction of polyacrylamide chains in the cross-linked graft copolymer, the values of water sorption and equilibrium water absorption increase (Table 2). A similar trend is valid for all our samples, regardless of the crosslinker introduction stage (Figure S4).

The crosslinker introduction stage significantly affects the sorption capacity of the copolymer, e.g., the lowest water absorption was observed for the XG-*g*-PAAm-1 (-4, -7) samples obtained by introducing MBA at the initiation stage (Table 2). The highest *W* values were achieved for XG-*g*-PAAm-3 (-6, -9), i.e., if the cross-linking agent was introduced at the chain termination stage. MBA introduction at the chain growth stage (XG-*g*-PAAm-2 (-5, -8) samples) also results in high *W* values, but the latter were somewhat lower compared to the XG-*g*-PAAm-3 (-6, -9) samples (*t_f_*). The difference in the *W* values of the samples obtained by adding MBA at the *t_i_* and *t_p_* stages (XG-*g*-PAAm-1 and -2, XG-*g*-PAAm-4 and -5, XG-*g*-PAAm-7 and -8) is almost leveled out as the grafting degree increases. In general, the ability of our graft copolymer to absorb water is significantly higher than is known in the literature [16,24]. The high sorption capacity of the graft copolymer is consistent with its highly developed surface (Figure 3) and the degree of crystallinity. Therefore, the determining factor in water absorption is the density and architecture of the 3D network of the graft copolymer.

To assess the physical parameters of our graft copolymer samples, the density (*ρ_c_*), proportion of the polymer in the swollen network of the graft copolymer (*f*) and the average (see above) molecular weight of interstitial regions (*M_c_*) were calculated. It was found that the values of *ρ_c_* and *f* decreased with an increase in the mass fraction of polyacrylamide in the graft copolymer, while *M*_c_ increased (Table 2). A decrease in the density of the air-dried copolymer and its proportion in the swollen state, as well as an increase in the length of the subchains between the crosslinks, was observed in the series *t_i_* → *t_p_* → *t_f_*, i.e., with an increase in the time of MBA introduction from the onset of the reaction. The higher the *M_c_* with equal contents of polyacrylamide and MBA, the less dense the resulting network structure and the higher the water absorption coefficient.

To perform a comparative analysis of hydration mechanisms in various aggregate states, the sorption behavior of the XG-g-PAAm graft copolymer with respect to saturated water vapor was studied. The Schroeder effect [41] was observed for this material, manifested in a significant excess of the amount of water sorbed in the liquid state over the sorption capacity in a saturated vapor atmosphere (Table 2). According to modern concepts, this effect, characteristic of ion-exchange polymers, may be associated with a kinetic ban on the penetration of the vapor sorbate phase into air-filled microvoids in the polymer sample, and the process itself occurs only on the accessible surface of the sorbent and at a lower rate than in the liquid [42]. In a liquid medium, water molecules penetrate the supramolecular structure due to both osmotic and hydrostatic pressure of the liquid, which leads to an increase in the volume of the copolymer and the availability of its microvoids and, consequently, to higher values of water sorption.

#### 3.3.2. Methylene Blue Sorption

It should be noted that the xanthan gum macromolecule contains carboxyl groups available for interaction with both the monomer and cross-linking agent. The possible participation and consumption of these groups in graft copolymerization may lead to a decrease in the sorption capacity of the polymeric substance for cationic compounds. To determine whether COOH groups participate in radical copolymerization, the sorption properties of our graft copolymer were studied using methylene blue as an example. As in the previous experiments, special attention was paid to the effect of the MBA introduction stage.

**Figure 5:**
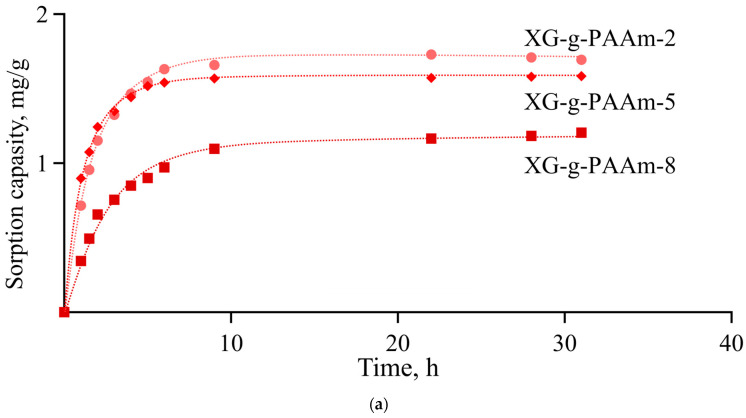

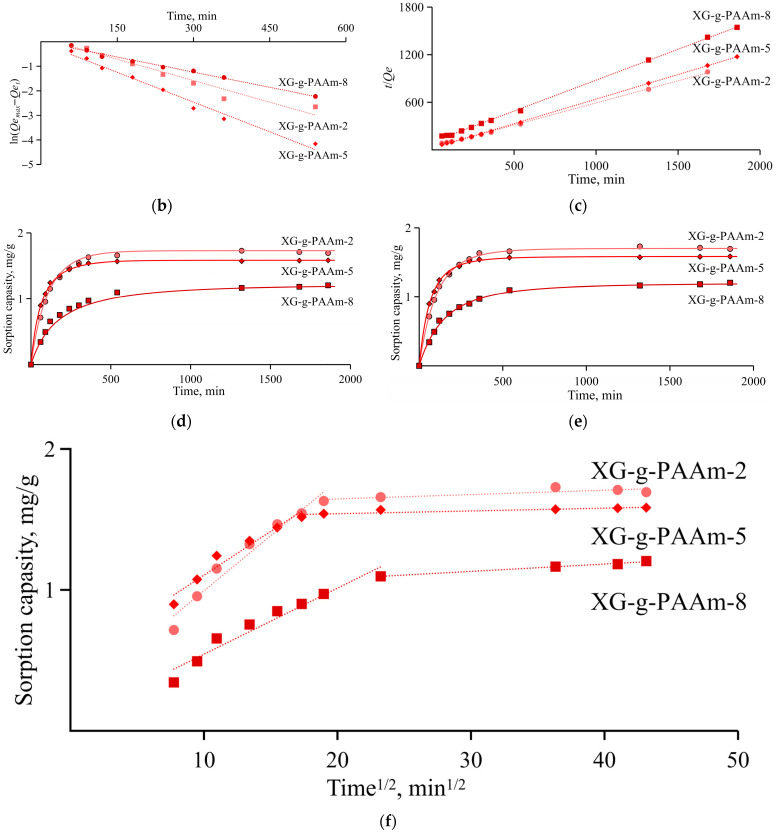
Kinetics of methylene blue sorption by XG-*g*-PAAm-2 (-5, -8) graft copolymer samples obtained by introducing the cross-linking agent at the chain termination stage (*t_p_*) in standard coordinates (**a**) and coordinates of pseudo-first (**b**) and pseudo-second order (**c**) models, the combined model (**d**) and the pseudo-*n*th order model (**e**) with theoretical *Qe* values (lines), as well as in the intraparticle diffusion coordinates (**f**).

The general form of the sorption kinetic curves is shown using XG-*g*-PAAm-2 (-5, -8) − *t_p_* samples with different degrees of grafting (Figure 5a). The kinetic curves for other graft copolymers are presented in the Supplementary Materials (Figure S5a,b) . As in the case of water sorption, intensive sorption of methylene blue by the copolymer occurred within 6–8 h. Equilibrium *Qe* values were reached after ~10–15 h of sorption. The sorption capacity of the graft copolymer for the ionic dye (unlike water) expectedly increases with a decrease in the grafting degree (Table 3). This trend is true for all samples with an increased *m*_XG_:*m*_AAm_ ratio, regardless of the *t_i_*, *t_p_* and *t_f_* stages. The MBA introduction stage also had little effect on the equilibrium *Qe* values of the cations. Keeping XG-*g*-PAAm − *t_i_* and XG-*g*-PAAm − *t_p_* in the dye solution, as well as in water, did not change the visual integrity of the copolymer. The exception was the XG-*g*-PAAm − *t_f_* samples, i.e., obtained by introducing MBA at the chain termination stage, for which a desorption branch was observed on the *Qe* = *f* (*t*) dependence due to their partial destruction.

Linearization of the kinetic curves of methylene blue sorption by our graft copolymer samples XG-*g*-PAAm-2 (-5, -8) − *t*_p_ using kinetic models (4) and (5) showed that the most accurate coincidence of the theoretical values of *Qe***′**_max_ with the experimental *Qe*_max_ was observed when using the pseudo-second-order model, *R*^2^ > 0.99 (Figure 5b,c, Table 3; Figure S5a,b). Checking the significance of the linear regression coefficients also showed that the same kinetic model was valid for an adequate description of the sorption kinetics (Table S1, Figure S5a,b). This indicates that chemisorption is the rate-limiting stage of the sorption process of the cationic dye. It is worth noting that the decimal order of the methylene blue sorption rate constant (*K*_2_) by our graft copolymer corresponds to that of some graft copolymers of a similar structure [7,19]. No significant effect of the grafting degree or the MBA introduction stage on *K*_2_ was found.

Until recently, pseudo-first and pseudo-second order kinetics calculations were considered alternative, and, as shown above, the one that best matched the experimental data was selected from the two results, and, accordingly, a conclusion was made about the sorption mechanism. However, Khamizov [40] showed that when conducting a kinetic experiment within a limited volume, when the sorbate consumption is significant, a quadratic term appears in the pseudo-first order equation, which is characteristic of the pseudo-second order, but not associated with chemisorption in this case. Therefore, it seems appropriate to use calculations according to the combined model Equation (9) (physical sorption within a limited volume) as an alternative, and also to vary the pseudo-order *n*, Equation (10), in order to identify whether the sorption process is approaching the first or second pseudo-order kinetics.

Our calculations using the combined model, Equation (9), and the sorption capacity per the polysaccharide component of the graft copolymer, Equation (10), showed that *Qe*_XG_ (with rare exceptions) increases with increasing AAm concentration in the reaction mixture and decreases with increasing time of MBA introduction from the start of the reaction (Table 3). This can be explained as follows. The carboxyl groups of xanthan gum are primarily responsible for methylene blue sorption. At high monomer concentrations and early introduction of the cross-linking agent, carboxyl groups are almost not involved in graft copolymerization. At the end of the reaction, they remain free, which leads to a high sorption capacity of the samples synthesized under such conditions. With low monomer concentrations and later introduction of the cross-linking agent, the probability of involving carboxyl groups in the copolymerization process increases, which, accordingly, switches them off from the subsequent sorption process.

**Figure 6:**
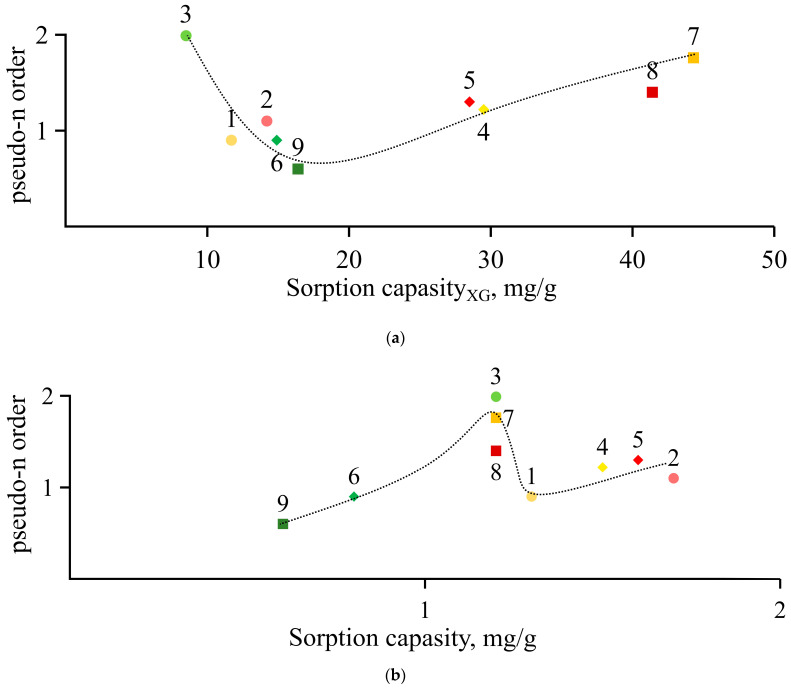
Correlation of the pseudo-nth order with the degree of methylene blue sorption: calculated per polysaccharide component (**a**) and experimental values (**b**); the numbers correspond to the sample number of XG-*g*-PAAm in Table 1.

An increase in *Qe*_XG_ naturally entails an increase in the pseudo-order *n* for the *t_i_* and *t_p_* stages of MBA introduction (Table 3), since the sorbate consumption becomes more and more significant, which, according to the combined model, entails an increase in the role of the quadratic term and a corresponding drift of *n* from 1 towards 2, not caused by the true pseudo-second order. An exception is the *t_f_* stage, i.e., the late introduction of the MBA. Here, the pseudo-order *n* increases from 0.6 (note that calculations according to Equation (9) for small *Qe*_XG_ give zero values of *K*_2_) up to 2 with a decrease in the AAm content and (in parallel) *Qe*_XG_. With such a small *Qe*_XG_ value as 8.5 mg/g (the smallest in Table 3), such a strong predominance of the quadratic term can no longer be explained by sorbate consumption (according to the combined model), and it should be recognized that it is caused precisely by the pseudo-second character of the sorption process. When the sorption degree is low, the sites with the maximum binding energy are filled first (pseudo-second-order chemisorptions). As the sorption degree (*Qe*_XG_) increases, the sites with lower binding energies also begin to fill, chemisorption is supplemented by physical sorption, and the calculated second order begins to drift towards unity. With further growth of *Qe*_XG_, sorbate consumption becomes noticeable, and the quadratic term increases again due to this. If we plot *n* vs. *Qe*_XG_, the pseudo-order for the XG-*g*-PAAm samples (*t_f_*) will fall on the descending branch, while for the XG-*g*-PAAm − *t_i_* and XG-*g*-PAAm samples (*t_p_*) it will fall on the ascending branch of the general dependence with a minimum (Figure 6a). The correlation of the pseudo-order of sorption with the experimental values of *Qe*_max_ behaves antibatically (Figure 6b).

Unfortunately, at this stage it seems impossible to split the Khamizov constant *K*_2_ into two terms corresponding to the pseudo-second order of chemisorption, on the one hand, and sorbate consumption, on the other hand (except for the indisputable case of XG-*g*-PAAm-3). This will be done later, after plotting the sorption isotherms and determining the maximum sorption degree of the copolymer.

Linearization of the kinetic sorption curves in the coordinates of the intraparticle diffusion model has shown the presence of a bend, indicating the occurrence of sorption both on the surface and in the bulk of the sorbent (Figure 5f). The first straight-line section reflects sorbate diffusion through the surface layer of the sorbent, the second—into the bulk of the graft copolymer particles. The diffusion inside the sorbent is thus not a process that completely controls sorption.

### 3.4. Model of the Spatial Architecture of the Graft Copolymer

**Figure 7:**
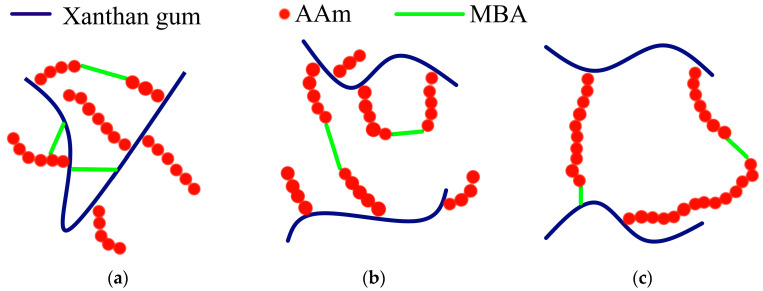
The most probable localizations of cross-linking nodes and internodal distances in the XG-*g*-PAAm graft copolymer synthesized under MW-assisted radical copolymerization conditions with introducing the cross-linking agent (MBA) at the stage of initiation (**a**), chain growth (**b**) or chain termination (**c**); *m*_AAm_ = const.

Based on our obtained experimental and theoretical data, the effect of the stage of introducing the cross-linking agent on the structure and sorption characteristics of the graft copolymer at *m*_AAm_ = const seems to be as follows. When introducing MBA at the initiation stage, when cross-linking nodes in the finished copolymer may form on the polyacrylamide branches and the main chain of xanthan gum, a chaotically cross-linked dense spatial network probably forms (Figure 7a). MBA introduction after the formation of (macro)radicals, i.e., at the stage of growth of side branches, leads to cross-linking of mainly growing polyacrylamide chains. This, in turn, is accompanied by the formation of a less dense network structure compared to the previous option, characterized by a smaller number of nodes and greater distances between them (Figure 7b). Finally, MBA introduction at the chain termination stage, when only the terminal fragments of the PAA kinetic chains and XG macroradicals are available for cross-linking, leads to the formation of a loose spatial network (Figure 7c). The number of crosslinks in the graft copolymer XG-*g*-PAAm − *t_f_* is less, and the internodal distance is significantly greater than in the XG-*g*-PAAm − *t_i_* and XG-*g*-PAAm − *t_p_* samples.

Our ideas about the differences in the spatial architecture of the XG-*g*-PAAm − *t_f_* (*t_i_*, *t_p_*) graft copolymer are consistent with their structural features, sample density, physicochemical parameters of the network and the average molecular weight of internodal regions, as well as sorption properties, e.g., the highest equilibrium water absorption was exhibited by the XG-*g*-PAAm − *t_f_* samples with the loosest spatial network. Next in descending order are XG-*g*-PAAm − *t_p_* and XG-*g*-PAAm − *t_i_*, whose density of the spatial structure increases in the series *t_p_* → *t_i_*. The effect of supramolecular ordering on the degree of crystallinity of the graft copolymer is not monotonous, as with water sorption, but is logically explainable. For the samples XG-*g*-PAAm − *t*_i_ and XG-*g*-PAAm − *t_f_*, obtained by introducing the cross-linking agent at the initiation or termination stage, low and close values of *χ* are characteristic. At the same time, the lower *W* values for XG-*g*-PAAm − *t*_i_ are due to the disordered supramolecular structure of the polymer network. The highest degree of crystallinity, but average *W* values, are characteristic of the samples XG-*g*-PAAm − *t_p_*, obtained by introducing MBA at the chain growth stage. This is explained by the homogeneity of the spatial framework and void cells in the bulk of the graft copolymer, which promotes directed diffusion of water molecules. High ordering of the XG-*g*-PAAm − *t_p_* polymer network is also confirmed by methylene blue sorption, which has shown the highest availability of functional fragments for participation in sorption for these samples.

The amount of acrylamide in the reaction system did not change the nature of the above-considered regularities of MW-assisted radical copolymerization. However, the higher the initial concentration of AAm, the longer the side branches and the distance between the cross-linking nodes in the samples, and, therefore, a looser network structure is formed. This is consistent with the increase in sorption and the diffusion coefficient of water of the XG-*g*-PAAm samples with an increase in the degree of grafting. An increase in the AAm concentration has virtually no effect on the availability of the functional groups of the copolymer participating in the cation-exchange process. A slight decrease in *Qe* values is observed only in the case of high concentrations of the acrylic monomer.

## 4. Conclusions

Samples of the cross-linked graft copolymer of xanthan gum with acrylamide with different degrees of grafting were synthesized, whose 3D spatial network differs in the localization and number of cross-linking nodes, internodal distance and the length of side branches. The study of their structure and properties has confirmed our hypothesis about the effect of the stage of introducing the cross-linking agent into the radical graft copolymerization reaction on the spatial architecture and properties of the samples synthesized, e.g., the samples obtained by introducing MBA at the chain termination stage, characterized by longer internodal sections of the network structure and a lower degree of crystallinity, exhibit the best water sorption capacity compared to those synthesized by introducing MBA at the initiation or chain growth stage. The unexpectedly discovered Schroeder effect (almost no water vapor sorption) also indicates the ionogenic structure of the synthesized graft copolymer. The samples obtained by introducing the cross-linking agent at the chain growth stage, when the spatial network is less loose compared to the later addition of MBA, but mainly polyacrylamide chains participate in the formation of spatial network nodes, exhibit the best sorption capacity for methylene blue.

Our study of the kinetics of methylene blue sorption by the synthesized samples, using not only the conventional pseudo-first and pseudo-second order models, but also the combined model and the average pseudo-order of sorption, has shown that the equilibrium degree of sorption (per xanthan gum) is higher, the higher the concentration of acrylamide in the reaction mixture and the earlier the MBA was introduced. A correlation curve “equilibrium sorption degree (per xanthan gum) vs. average pseudo-order of sorption” was plotted, which passes through a minimum. It is assumed that chemisorption (ionic binding) occurs at low fillings of the sorption sites and the effect of sorbate consumption at high fillings of these sites.

Thus, the discovered effect of the degree of grafting and the MBA introduction stage on the structure and properties of the cross-linked graft copolymers of xanthan gum with acrylamide opens up prospects for the targeted synthesis of not only selective sorbents for specific sorption tasks, but also specific proton-exchange membranes of new functional significance.

## Figures and Tables

**Table 1:** Quantitative characteristics of the synthesis of graft copolymer XG-*g*-PAAm.

Sample No. XG-g-PAAm	Volume of AAm Solution, mL	Mass Ratio *m*_XG_:*m*_AAm_	MBA Introduction Stage	Degree of Grafting *G*, %
1	2	0.12:0.8	*t_i_*	790 ± 40
2			*t_p_*	735 ± 70
3			*t_f_*	610 ± 55
4	4	0.12:1.6	*t_i_*	1800 ± 175
5			*t_p_*	1690 ± 195
6			*t_f_*	1655 ± 210
7	8	0.12:3.2	*t_i_*	3590 ± 260
8			*t_p_*	3350 ± 300
9			*t_f_*	2880 ± 330

**Table 2:** Physicochemical characteristics of the graft copolymer samples.

Sample No. XG-*g*-PAAm	Degree of Crystallinity *χ*, %	Degree of H_2_O Sorption		Physical Parameters of Graft Copolymer		
		Liquid *W_im_*, g/g	Vapor *W_vap_*, g/g	*ρ_c_*, g/cm^3^	*f*	*M_c_*, g/mol
1	26.5	8.14 ± 0.31	0.29	1.2298	0.351	700
2	30.5	9.45 ± 0.58	0.19	1.1775	0.302	1130
3	26.7	10.31 ± 0.46	0.04	1.1039	0.277	1430
4	29.1	10.23 ± 0.69	0.20	1.2722	0.279	1320
5	47.9	10.71 ± 0.52	0.09	1.1530	0.267	1380
6	39.4	12.26 ± 0.33	0.19	1.1588	0.233	2115
7	24.2	12.96 ± 0.67	0.18	1.1530	0.220	2230
8	27.7	13.17 ± 0.57	0.11	1.1180	0.217	2265
9	27.9	17.16 ± 0.93	0.22	1.1131	0.167	4830

**Table 3:** Physicochemical parameters of methylene blue sorption by the graft copolymer XG-*g*-PAAm: experimental ones and those calculated using the kinetic models of pseudo-first (4) and pseudo-second order (5), the combined model (9) and pseudo-*n*th order (10).

Sample XG-*g*-PAAm	*Qe_max_* *, mg/g	Pseudo-First Order			Pseudo-Second Order			Combined Model		Pseudo-*n*th Order		*Qe*_XG_, mg/g
		*Qe*′*_max_*, mg/g	*K*_1_∙10^2^, min^−1^	*R* ^2^	*Qe*′*_max_*, mg/g	*K*_2_, g/(mg·min)	*R* ^2^	*K*_1_∙10^2^, min^−1^	*K*_2_∙10^1^, g/(mg·min)	*K*∙10^2^	*n*	
1	1.3	1.4	0.6	0.9879	1.5	0.01	0.9962	0.6	~0	0.6	0.9	11.7
2	1.7	1.5	0.7	0.9487	1.8	0.01	0.9991	0.8	0.01	8.9	1.2	14.2
3	1.2	0.5	0.9	0.9764	1.2	0.03	0.9995	0.2	0.30	2.6	2.0	8.5
4	1.5	1.4	0.8	0.9652	1.6	0.01	0.9992	0.6	0.03	0.9	1.2	29.5
5	1.6	1.2	0.9	0.9709	1.6	0.02	0.9998	0.7	0.10	1.4	1.3	28.5
6	0.8	0.9	0.8	0.9827	1.2	0.01	0.9524	0.6	~0	0.6	0.9	14.9
7	1.2	0.9	0.5	0.9517	1.2	0.01	0.9992	0.2	0.10	1.0	1.8	44.3
8	1.2	0.7	0.3	0.9905	1.3	0.01	0.9992	0.2	0.04	0.6	1.4	41.4
9	0.6	0.8	1.0	0.9726	0.8	0.01	0.9664	0.7	~0	0.4	0.6	16.4

* The error in determining *Qe_max_* was 0.002–0.006 mg/g.

## Data Availability

The original contributions presented in the study are included in the article/Supplementary Materials, further inquiries can be directed to the corresponding authors.

## Supplementary Materials

The following supporting information can be downloaded at: https://www.mdpi.com/article/10.3390/polym17212841/s1, Table S1: Quantitative characteristics of the synthesis process of graft copolymer XG-g-PAAm; Figure S1: FTIR spectra of the XG-g-PAAm-1 (-2, -3, -7, -8, -9) copolymer samples. The dotted lines highlight the vibration ranges of the main characteristic signals: *1*—υ_O-H_, υ_N-H_; *2*—υ_C-H_, δ_C-H_; *3*—υ_-COO-_, _C=O_; *4*—υ_C-N_, δ_C-H_; *5*—υ_C-O, C-C, C-H_.; Figure S2： (a) X-ray diffraction patterns of xanthan gum and XG-g-PAAm-1 (-2, -3) samples obtained at a mass ratio of mXG:mAAm 0.12:0.8. The amorphous halo is indicated by the purple lines.; (b) X-ray diffraction patterns of xanthan gum and XG-g-PAAm-7 (-8, -9) samples obtained at a mass ratio of mXG:mAAm 0.12:3.2. The amorphous halo is indicated by the purple lines.; Figure S3: Sorption kinetics of liquid water and H2O vapor (marked with a prime) by XG-g-PAAm-1 (-4, -7) graft copolymer samples obtained by introducing the cross-linking agent at the chain termination stage (*t_i_*) (a), by XG-g-PAAm-2 (-5, -8) graft copolymer samples obtained by introducing the cross-linking agent at the chain termination stage (*t_p_*) (b).; Figure S4: Kinetics of methylene blue sorption by XG-g-PAAm-1 (-4, -7) graft copolymer samples obtained by introducing the crosslinking agent at the stage of chain termination (*t_i_*) (a), by XG-g-PAAm-3 (-6, -9) graft copolymer samples obtained by introducing the crosslinking agent at the stage of chain termination (*t_f_*) (b) in standard coordinates.; Figure S5: (a) Kinetics of methylene blue sorption by XG-*g*-PAAm-1 (-4, -7) graft copolymer samples obtained by introducing the crosslinking agent at the stage of chain termination (*t_i_*) in coordinates of pseudo-first (A) and pseudo-second order (B) models, the combined model (C) and the pseudo-*n*th order model (D) with theoretical *Qe* values (lines).; (b) Kinetics of methylene blue sorption by XG-*g*-PAAm-3 (-6, -9) graft copolymer samples obtained by introducing the crosslinking agent at the stage of chain termination (*t_f_*) in coordinates of pseudo-first (A) and pseudo-second order (B) models, the combined model (C) and the pseudo-*n*th order model (D) with theoretical *Qe* values (lines).; Figure S6: Kinetics of methylene blue sorption by XG-*g*-PAAm-1 (-4, -7) graft copolymer samples obtained by introducing the crosslinking agent at the stage of chain termination (*t_i_*) (a) and by XG-*g*-PAAm-3 (-6, -9) graft copolymer samples obtained by introducing the crosslinking agent at the stage of chain termination (*t_f_*) (b) in the coordinates of intraparticle diffusion.; Table S2. The significance of linear regression coefficients of the kinetics of the sorption of methylene blue by XG-g-PAAm samples in the coordinates of kinetic models.

## Abbreviations

The following abbreviations are used in this manuscript:

XG	Xanthan gum
AAm	Acrylamide
MBA	*N*,*N*-methylene*bis*acrylamide
XG-g-PAAm	Graft-copolymer of xanthan gum with acrylamide
MW	Microwave
