# Supplementary material for: Effect of the Crosslinker Introduction Stage on the Structure and Properties of Xanthan Gum–Acrylamide Graft Copolymer
Source: Polymers (Basel). 2025 Oct 24;17(21):2841. doi: 10.3390/polym17212841 (PMC12609235; doi:10.3390/polym17212841)
Supplement: Supplementary file 1 [file polymers-17-02841-s001.zip › polymers-3882613-supplementary.pdf]

Effect of the crosslinker introduction stage on the structure and properties of xanthan gum–acrylamide graft copolymer

Anton K. Smirnov, Diana F. Pelipenko, Sergey L. Shmakov, Andrey M. Zakharevich and Anna B. Shipovskaya

Supplementary Materials

Table S1. Quantitative characteristics of the synthesis process of graft copolymer XG-g-PAAm

Sample No. XG-*g*-PAAm	Volume of AAm solution, ml	Mass ratio $m_{XG}:m_{AAm}$	MBA introduction stage
1	2	0.12:0.8	t_i
2			t_p
3			t_f
4	4	0.12:1.6	t_i
5			t_p
6			t_f
7	8	0.12:3.2	t_i
8			t_p
9			t_f

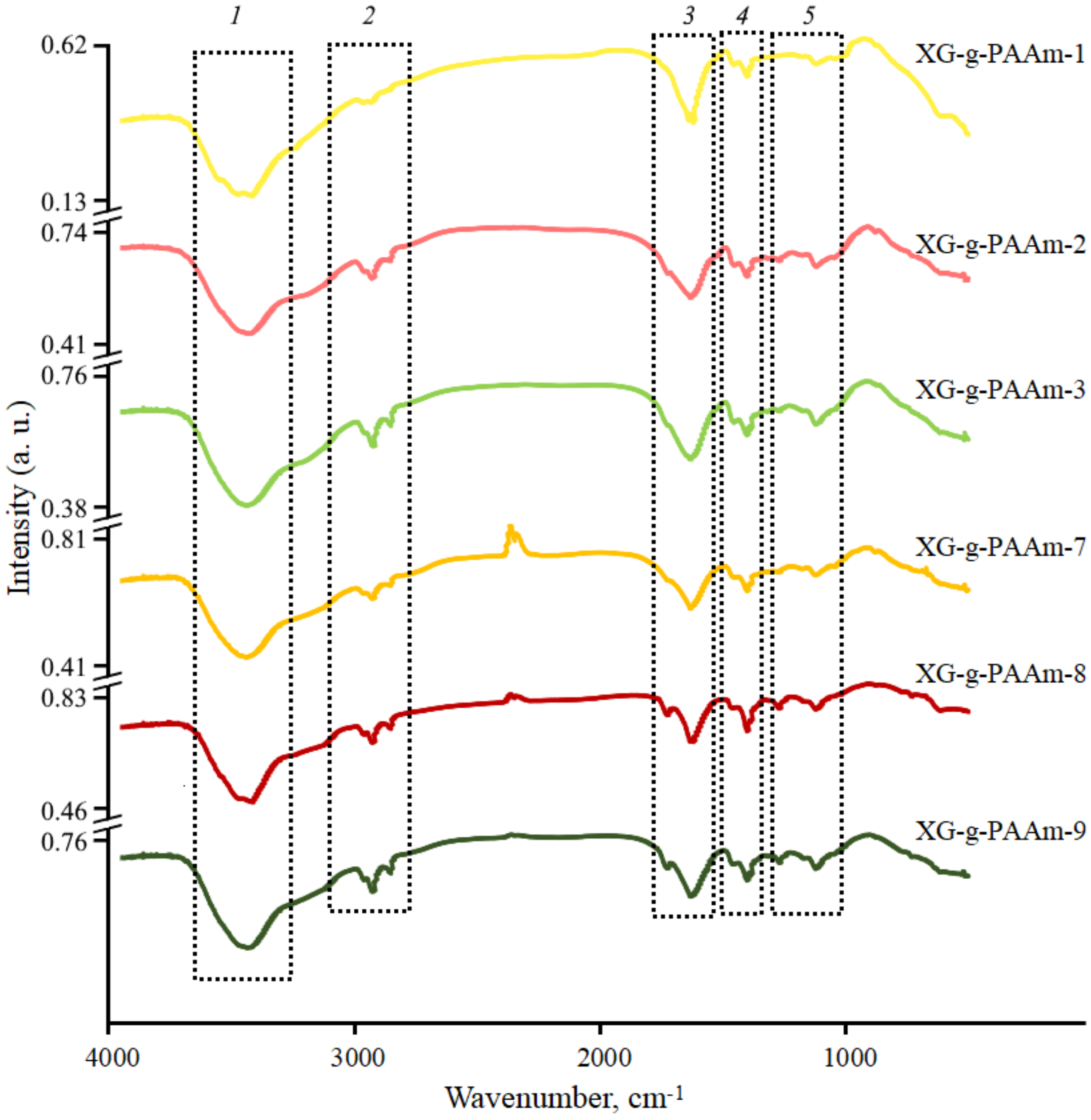

Figure S1. FTIR spectra of the XG-g-PAAm-1 (-2, -3, -7, -8, -9) copolymer samples. The dotted lines highlight the vibration ranges of the main characteristic signals: *1* – $\upsilon_{O\text{-}H}$, $\upsilon_{N\text{-}H}$; *2* – $\upsilon_{C\text{-}H}$, $\delta_{C\text{-}H}$; *3* – $\upsilon_{\text{-}COO\text{-},\ C=O}$; *4* – $\upsilon_{C\text{-}N}$, $\delta_{C\text{-}H}$; *5* – $\upsilon_{C\text{-}O,\ C\text{-}C,\ C\text{-}H}$.

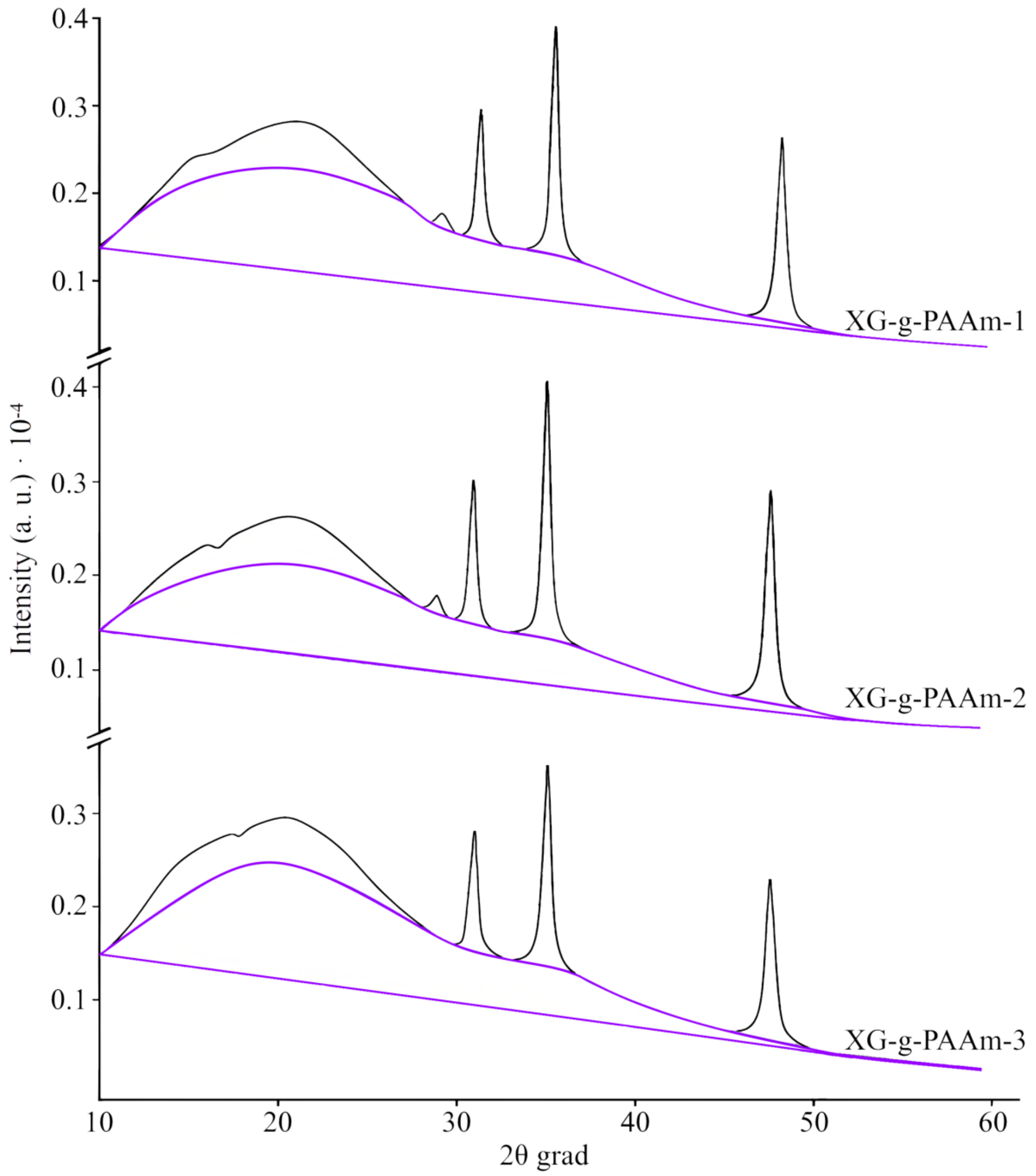

Figure S2a. X-ray diffraction patterns of xanthan gum and XG-g-PAAm-1 (-2, -3) samples obtained at a mass ratio of mXG:mAAm 0.12:0.8. The amorphous halo is indicated by the purple lines.

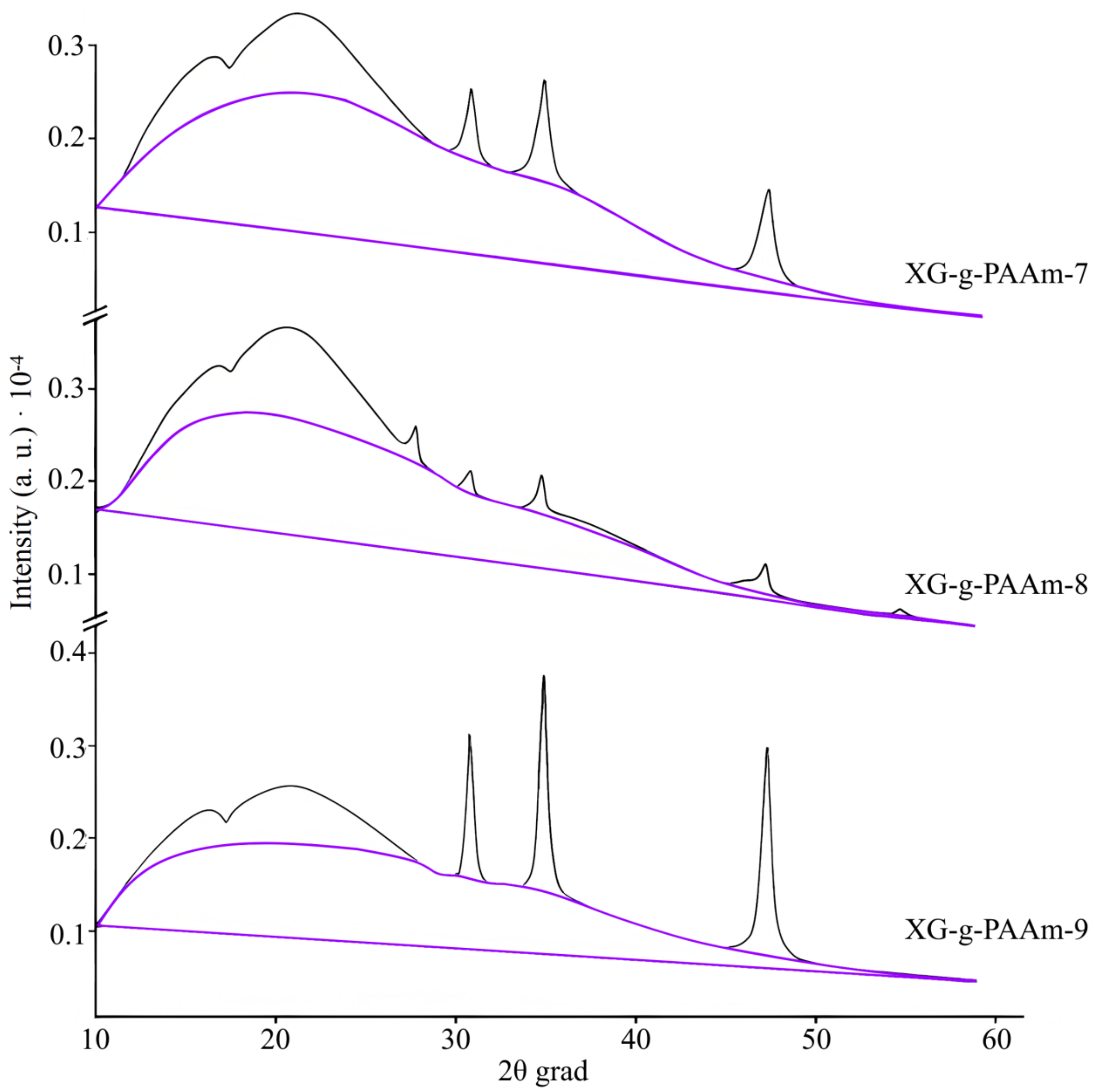

Figure S2b. X-ray diffraction patterns of xanthan gum and XG-g-PAAm-7 (-8, -9) samples obtained at a mass ratio of mXG:mAAm 0.12:3.2. The amorphous halo is indicated by the purple lines.

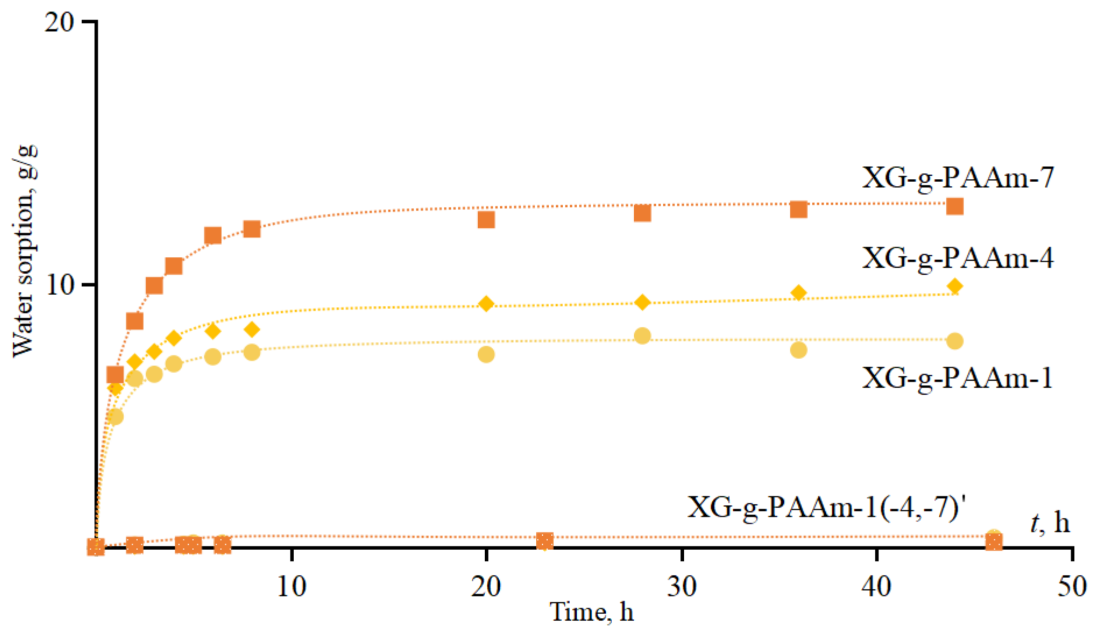

(a)

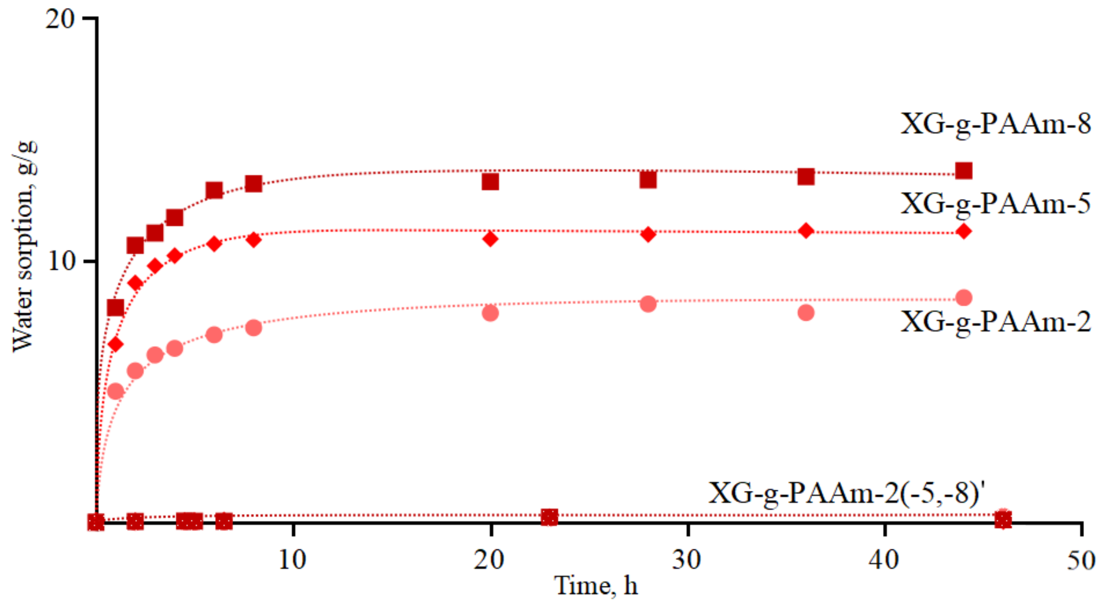

(b)

Figure S3. Sorption kinetics of liquid water and H2O vapor (marked with a prime) by XG-g-PAAm-1 (-4, -7) graft copolymer samples obtained by introducing the cross-linking agent at the chain termination stage (t_i) (a), by XG-g-PAAm-2 (-5, -8) graft copolymer samples obtained by introducing the cross-linking agent at the chain termination stage (t_p) (b).

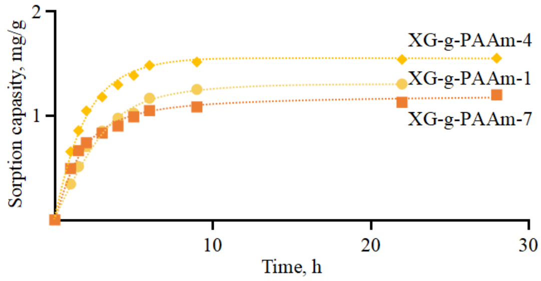

(a)

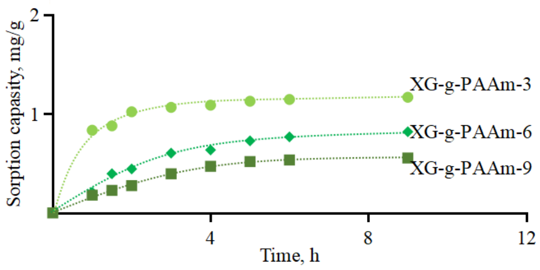

(b)

Figure S4. Kinetics of methylene blue sorption by XG-g-PAAm-1 (-4, -7) graft copolymer samples obtained by introducing the crosslinking agent at the stage of chain termination (t_i) (a), by XG-g-PAAm-3 (-6, -9) graft copolymer samples obtained by introducing the crosslinking agent at the stage of chain termination (t_f) (b) in standard coordinates

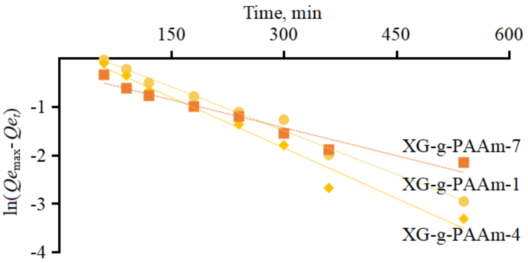

(a)

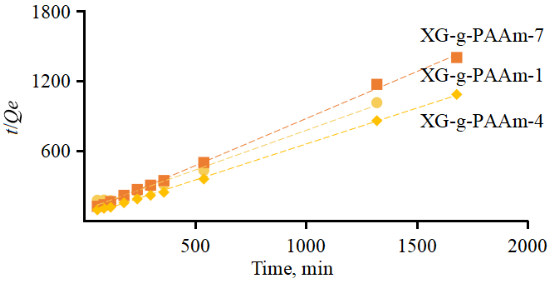

(b)

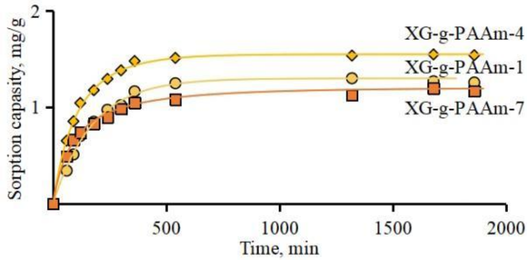

(c)

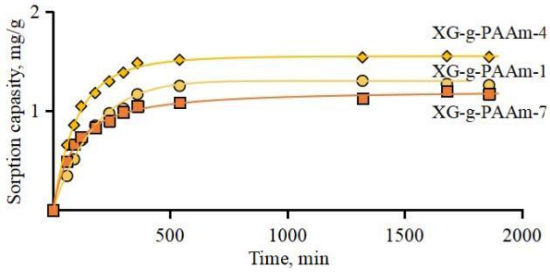

(d)

Figure S5a. Kinetics of methylene blue sorption by XG-*g*-PAAm-1 (-4, -7) graft copolymer samples obtained by introducing the crosslinking agent at the stage of chain termination (t_i) in coordinates of pseudo-first (a) and pseudo-second order (b) models, the combined model (c) and the pseudo-n^{th} order model (d) with theoretical *Qe* values (lines).

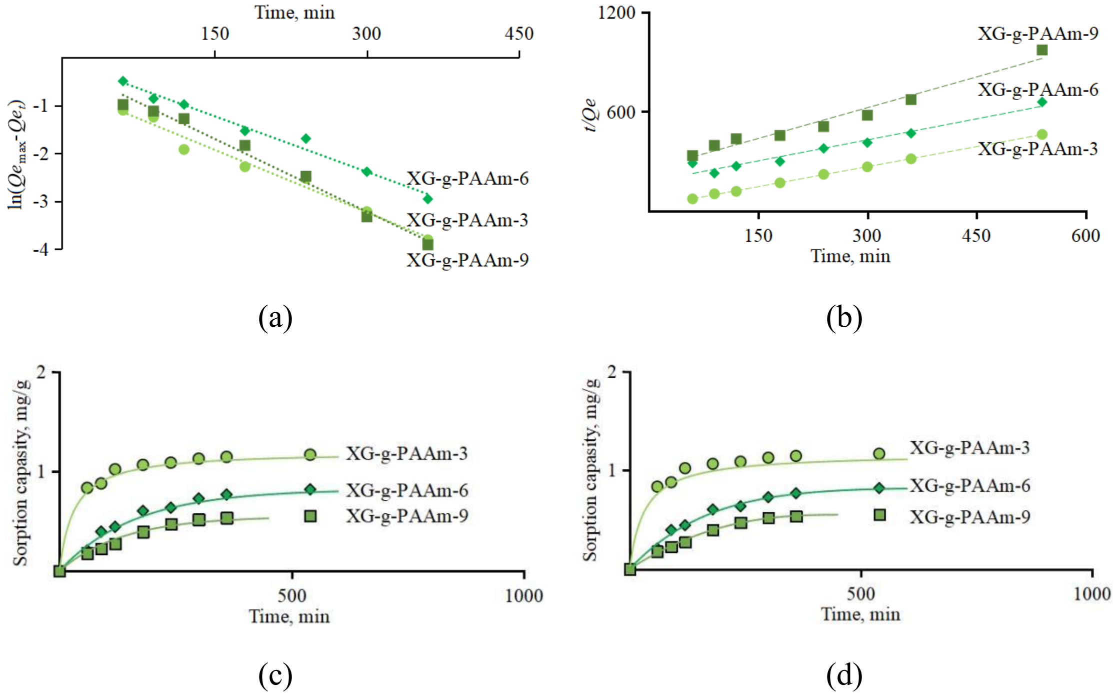

Figure S5b. Kinetics of methylene blue sorption by XG-*g*-PAAm-3 (-6, -9) graft copolymer samples obtained by introducing the crosslinking agent at the stage of chain termination (t_f) in coordinates of pseudo-first (a) and pseudo-second order (b) models, the combined model (c) and the pseudo-n^{th} order model (d) with theoretical Qe values (lines).

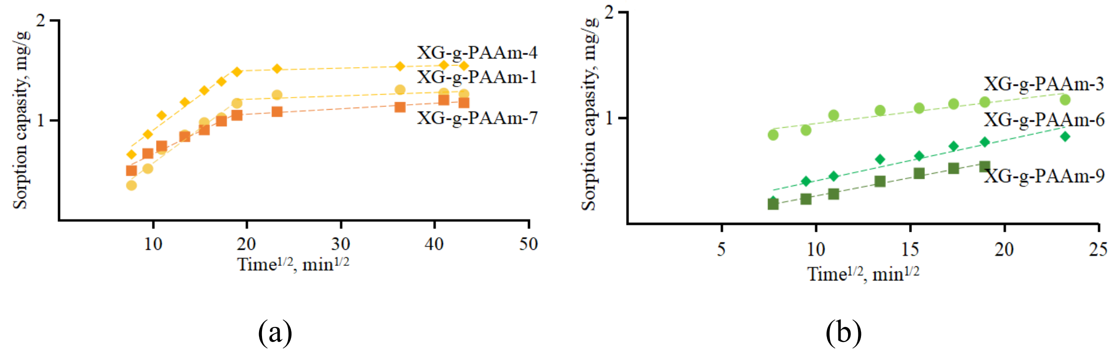

Figure S6. Kinetics of methylene blue sorption by XG-*g*-PAAm-1 (-4, -7) graft copolymer samples obtained by introducing the crosslinking agent at the stage of chain termination (t_i) (a) and by XG-*g*-PAAm-3 (-6, -9) graft copolymer samples obtained by introducing the crosslinking agent at the stage of chain termination (t_f) (b) in the coordinates of intraparticle diffusion.

Table S2. The significance of linear regression coefficients of the kinetics of the sorption of methylene blue by XG-g-PAAm samples in the coordinates of kinetic models

Sample No. XG-*g*-PAAm	y=(b±Δb)x + (a±Δa)	
	Pseudo-first order	Pseudo-second order
1	(-0.006±0.00064)x + (0.3013±0.17930)	(0.6818±0.05)x+(93.233±27.00)
2	(-0.006±0.0013)x + (0.167±0.3674)	(0.5576±0.01)x + (34.273±10.47)
3	(-0.0087±0.00051)x + (–0.712±0.15396)	(0.809±0.02)x + (24.258±4.65)
4	(-0.0069±0.0011)x + (0.2193±0.3056)	(0.6192±0.0179)x+(38.287±12.8673)
5	(-0.0081±0.0011)x – (0.035±0.3044)	(0.62±0.0074)x+(19.626±6.5285)
6	(-0.0077±0.0011)x – (0.0681±0.2463)	(0.8488±0.1556)x + (174.54±43.5719)
7	(-0.0038±0.0081)x – (0.2905±2.2625)	(0.8096±0.0228)x+(66.543±16.4167)
8	(-0.0041±0.0004)x – (0.0013±0.1097)	(0.7791±0.0161)x+(99.701±14.2984)
9	(-0.0102±0.0014)x – (0.1722±0.2994)	(1.2432±0.1830)x + (250.8±45.84)
